# A systematic review of the implementation of cancer-specific holistic needs assessment (HNA) in adult clinical practice, and applicability to the brain tumour population

**DOI:** 10.1007/s00520-026-10609-x

**Published:** 2026-04-02

**Authors:** Stephanie Sivell, †Timothy Hamilton, Mala Mann, Elin Baddeley, Helen Bulbeck, Ameeta Retzer, Kathy Seddon, Anthony Byrne

**Affiliations:** 1 Marie Curie Research Centre, Division of Population Medicine, School of Medicine, Cardiff University, 3rd Floor, Neuadd Meirionnydd, Heath Park, Cardiff, CF14 4YS UK; 2 Aneurin Bevan University Health Board, Newport, UK; 3 Specialist Unit for Review Evidence, Cardiff University, Cardiff, UK; 4 brainstrust – the brain cancer people, Cowes, Isle of Wight, UK; 5 Centre for Patient Reported Outcomes Research, Department of Applied Health Sciences, University of Birmingham, Birmingham, UK; 6 National Institute of Health and Care Research (NIHR) Applied Research Collaboration West Midlands, Centre for Evidence and Implementation Science, University of Birmingham, Birmingham, UK; 7 Velindre Cancer Centre, Velindre University NHS Trust, Cardiff, UK; 8 Marie Curie Hospice, Cardiff and the Vale, Penarth, UK

**Keywords:** Cancer, Brain cancers, Glioma, Needs assessment, Implementation science, Systematic reviews

## Abstract

**Purpose:**

This review aimed to identify and review any brain tumour-specific holistic needs assessment (HNA) and the clinical implementation of any HNAs in an adult cancer population.

**Methods:**

A systematic review and narrative synthesis. Five electronic databases were searched from 2008 to 2023 and updated in January 2025. Reference lists of systematic reviews were screened for relevant studies. Two independent reviewers performed study selection, critical appraisal, and data extraction.

**Results:**

Four HNA tools implemented in clinical practice, across 9 studies, were included. One study was adapted for brain tumour patients. Evidence of widespread implementation in clinical practice was limited and varied evidence, relating to the timing, location and mode of delivering. Patients were more likely to report on physical rather than psychosocial needs, with limited evidence of patient and staff satisfaction with the use of HNA.

**Conclusion:**

There is limited and varied evidence of HNAs being successfully implemented in brain tumour and the wider cancer healthcare settings. Tailored interventions for the needs of people with brain tumour have the potential to address the complexities this population faces. However, clear evidence-based guidance to develop relevant HNAs for successful implementation in clinical settings is needed.

## Introduction

Brain tumours present a significant global burden and are an important public health issue worldwide [1]; the estimated age-standardised incidence rate of central nervous system (CNS) cancer is 4.63 per 100 000 person-years [2]. Based on Global Cancer Observatory (GLOBOCAN) 2020 estimates, brain and CNS cancer account for 1.9% of all cancer diagnoses and are responsible for 2.5% of all cancer deaths [3]. The needs of patients with brain tumours are unique and complex. Unlike other cancers, the brain cancer trajectory encompasses significant cognitive impairment and neuro-behavioural changes as well as progressive physical symptoms [4]. Furthermore, brain tumour patients report poorer patient experiences than in any other cancer group [5]. The unmet needs of those with cancer are increasingly recognised [6], emphasising the need to enhance care to address unmet needs and improve quality of life [7]. A specific holistic needs assessment (HNA) for people with brain tumours would identify and facilitate intervention to address unmet needs [8]. We reviewed the implementation of cancer-specific HNA in adult clinical practice, and their general applicability to the brain tumour population.

The long-term consequences of living with glioma include living with uncertainty long after diagnosis and treatment, prognostic uncertainty, and anxiety surrounding recurrence and worsening symptoms [8]. Frequently reported symptoms for brain tumours include headache, personality change, confusion, weakness, reduced mobility and gait disturbance, language difficulties, mood disturbance, poor concentration, and reduced mental capacity [9, 10]. Seizures are common and can have a devastating impact on patients’ employment and independence [11]. Epilepsy and its treatments can exacerbate neuropsychological and physical problems, negatively impacting quality of life [12]. There is also a substantial burden placed on family caregivers of patients diagnosed with brain tumours. Caregivers of patients with brain tumours report significantly higher levels of strain and lower levels of mental wellbeing when compared to those of other cancer groups [13]. Evidence suggests that caregivers experiencing financial difficulties had significantly higher odds of reporting multiple ‘high/very high’ unmet needs [13].

HNA is a structured approach to bring together information across all domains of care, including the physical, psychological, social and spiritual care needs [14] informing a comprehensive, holistic and patient-centred care plan in oncology settings [15] and may include specific measures. There are numerous tools that are commonly used for HNA across all cancer types, including: the Sheffield Profile for Assessment and Referral to Care (SPARC) [16]; National Comprehensive Cancer Network distress thermometer (NCCN DT) [17] and concerns checklist; the Macmillan electronic HNA (eHNA) [18]; the Palliative Care Needs Assessment Tool (PC-NAT) [19]; the Liverpool Patients Concerns Inventory (PCI) [20]. Tools for HNA are typically delivered as a questionnaire during a clinical interaction between a patient and a healthcare professional, or remotely by a patient, and can be delivered in a paper format or electronically [21]. HNA, along with care planning should be used at key points in the cancer patient’s trajectory; at diagnosis, towards the end of treatment, and whenever patients’ needs change or when requested by the patient, to enable a developed care plan which focuses on the most pertinent concerns for the patient [21].

Research investigating the overall effectiveness of using such tools to screen for distress is not yet conclusive, but screening appears to improve communication between patients and clinicians and may enhance psychosocial referrals [22]. HNAs have been found to change the dynamics of clinical consultations away from being clinician-led towards shared decision-making [23]. These tools have been validated for use in general cancer populations [24] and have been shown to identify unmet needs in certain cancer populations [25]. However, due to the particular impact on communication, cognitive and social function, these general checklists may not be suitable in assessing the specific needs of individuals with brain tumours [26, 27]. A systematic review identified four potential HNA tools for use in brain cancer, though found that none had strong psychometric properties, demonstrating the need to further develop tools to optimise HNA as a meaningful intervention [28].

To generate evidence of relevance to the brain tumour population, we undertook a systematic review of the implementation and impact of HNA for people affected by cancer. We aimed to:

1. Identify if there are any pre-existing brain tumour specific HNAs being routinely used in clinical practice
2. Review evidence of the clinical implementation of an HNA in any adult cancer population.

## Methods

The systematic review is reported according to Preferred Reporting Items for Systematic Reviews and Meta-Analyses 2020 (PRISMA 2020) [29]. The review protocol was registered with PROSPERO (CRD42023434077). The review was conducted in accordance with Palliative Care Evidence Review Service (PaCERS) modified systematic review methodology [30]. Our original aim was to carry out a rapid review. However, due to the scarcity of the identified literature, we decided to progress with undertaking a systematic review, expanding to include evidence of HNA in cancer in any adult cancer population.

### Search strategy

A comprehensive search was carried out across five databases: Ovid Medline All (which includes In-Process and other Non-Indexed Citations), Ovid Embase, Ovid Emcare, Ovid PsycINFO and Elsevier Scopus. These databases were selected to capture the broad range of study designs, interventions and outcomes relevant to our review. Ovid Medline provides extensive international biomedical coverage, including allied health and related sciences, and Cardiff University’s subscription includes in-process and ‘ahead of print’ citations. Pubmed was not used because it primarily provides another route to Medline, which would have duplicated our search. Ovid Embase offers additional biomedical coverage, including trials and conference abstracts. Ovid Emcare focuses on nursing and allied health professions, removing the need to search CINAHL. Ovid PsycINFO covers psychology, social, behavioural, and health sciences supporting our interest in holistic needs. Finally, Elsevier Scopus adds broader interdisciplinary coverage across life, health, physical, and social sciences.

**Fig. 1:**
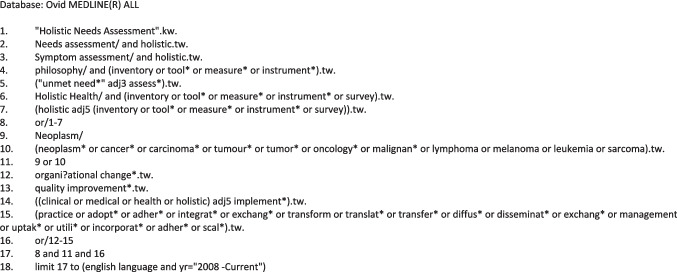
MEDLINE search strategy

The search strategy was developed in Ovid Medline using a combination of text words and Medical Subject Headings, with dates limited to January 2008–14 March 2023 and updated in January 2025 (Fig. 1). Furthermore, we searched websites of three relevant organisations (Macmillan Cancer Support, Marie Curie and NCRI) and reference lists of systematic reviews were screened for appropriate studies. We included studies which reported on adults diagnosed with cancer within any Organisation for Economic Co-operation and Development (OECD) countries [31] to ensure comparability across healthcare systems which have health care economies comparable to the UK, an HNA to identify concerns and needs of people with cancer and outcomes which may address patient participation and/or shared decision-making and implementation of the HNA; the HNA would typically include domains such as physical, psychological or emotional, spiritual, social, practical and other domains of needs to provide a complete assessment which can aid in planning appropriate supportive care (see Table 1). All types of study design which included a component of implementation were also included (see Table 1 for full details). All identified references were uploaded to a reference manager software EndNote X20.6 [32] and screened by the one reviewer (MM) for duplicates. The final deduplicated EndNote library was imported into Rayyan [33], and screening was conducted by two independent review authors. Eligibility criteria were used to assess the titles and abstracts, and then full text of all sources identified by the search. The study selection was conducted independently by all reviewers. Review authors were involved in study selection (AB, EB,TH,MM,AR,SS) and disagreements were resolved through discussions.

**Table 1:** Eligibility criteria of included studies

Inclusion criteria	Exclusion criteria
Adults diagnosed with cancer within Organisation for Economic Co-operation and Development (OECD) countries.	Individuals < 18, from non-OECD (Organisation for Economic Co-operation and Development) countries and/or non-malignant conditions.
Interventions used to identify concerns and unmet needs in cancer, often referred to as holistic needs assessment (HNA).	Interventions which do not identify concerns and unmet needs in cancer, or which do not have a focus on the implementation of an HNA intervention.
There were no restrictions on comparisons; all included groups or interventions could be compared with each other, and a separate control group was not required.	There were no restrictions on comparisons; all included groups or interventions could be compared with each other, and a separate control group was not required.
Outcomes refer to patient participation and shared decision making.	Any outcomes which do not refer to patient participation or shared decision making.
Implementation outcomes such as acceptability (patients, caregivers, clinicians), completion, organisational adoption, appropriateness, feasibility, fidelity, implementation cost, penetration and sustainability	Outcomes which do not refer to implementation of the intervention such as acceptability (patients, caregivers, clinicians), completion, organisational adoption, appropriateness, feasibility, fidelity, implementation cost, penetration and sustainability.
All types of study design that include an implementation component.	Protocol papers, opinion pieces, theory papers and conference proceedings where only an abstract was available) were excluded.

### Data extraction

Review authors completed data extraction independently for each study. A standard data extraction form was created and pilot-tested to ensure reliability and comprehensiveness of the data extraction process. Any discrepancies between reviewers were resolved through discussion or by consulting a third reviewer, ensuring that the final data was reliable and consistent across the study.

### Methodological quality assessment

Quality assessment was conducted on all included studies using the appropriate checklist from the Specialist Unit for Review Evidence (SURE) [34]. Quality assessment was completed by one review author and checked against the original article by a second review author for accuracy. Any disagreements were resolved through discussion. These assessments were used to indicate reliability, consistency, and relevance of the evidence.

### Data synthesis

Due to the expected heterogeneity of the evidence, we planned to undertake a detailed narrative of the results**.** The PaCERS methodology was extended to include a narrative synthesis method, which integrated and described the results of the included studies [35]. Research Partner and advocacy (KS, HB) reviewed and supported the interpretation of the synthesis.

## Results

### Study characteristics

**Fig. 2:**
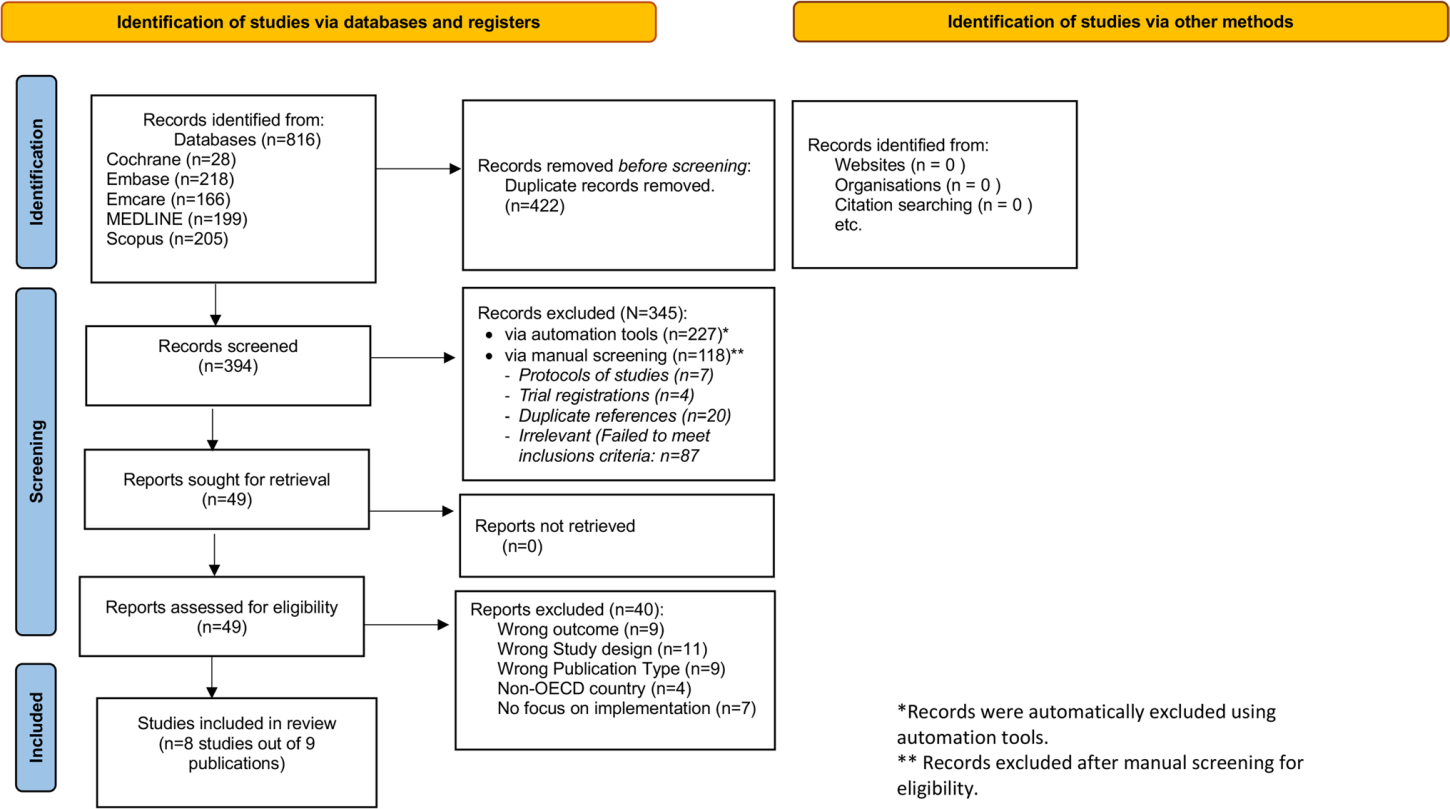
PRISMA flow diagram

The process of study retrieval is reported in the PRISMA 2020 flow diagram (Fig. 2) [29]. 816 studies were identified, 394 abstracts were screened, and 49 papers retrieved for full text screening. Of those full text papers, nine studies were eligible for the review of which eight were undertaken in the UK [23, 36–42] and one study was undertaken in Australia [43]. Four tools to address HNAs in cancer were identified [23, 36–43] of which one study was specifically aimed for brain tumour patients [40]. Characteristics of the included studies are presented in Table 2 comprising an overview of the following: identified HNAs; study design location and aims; cancer types and participants; HNA format and purpose.

**Table 2:** Study and HNA characteristics

HNA	Study, location, and design	Study aims	Cancer type and participants	HNA format and purpose
The Liverpool Patients Concerns Inventory (PCI)	** Rooney et al. 2014** Edinburgh, Scotland Cross-sectional and qualitative analyses	To develop and publicise a brain tumour PCI, to report common concerns reported among patients participating in its initial study and to report specific questions asked by patients regarding their care.	Brain Adult patients (age ≥ 18 years) with an intracranial tumour 77 patients eligible; *n* = 53/77 completed the questionnaire.	• PCI adapted for specific use in neuro-oncology outpatients’ clinic. • Information leaflet and ‘initial feedback’ form posted to patients before their clinical appointment and to be completed before clinic. • Feedback form asked: Ø how long it took to complete the PCI; Ø who completed the PCI; Ø how hard it was to complete the PCI; Ø any concerns should be added to the checklist.
	** Ghazali et al. 2011** Liverpool, England Cross sectional study in the Merseyside Regional Maxillofacial Unit	To compare the referral trends following consultation in the time periods before and after introduction of PCI in an oncology outpatient clinic.	Head and neck 247 patients at least cancer free 6 weeks post-treatment.	• Self-completed questionnaires using a Microsoft Access form through a standardised touch screen computer. • PCI used in the waiting room to screen patients’ needs/concerns before the outpatient’s consultation.
Macmillan’s Holistic Needs Assessment (HNA)	** Briggs et al. 2020; 2023 ** Nottingham, England Semi-structured Interviews in Nottingham University NHS Trust (2020) Qualitative case study using semi-structured interviews across four hospitals in England (2023)	To understand the women’s experiences of having breast cancer, and of completing Macmillan’s eHNA as part of their care (2020). To explore views of HNAs from the perspectives of healthcare professionals and women with breast cancer, including how the HNA contributed to providing support (2023).	Breast 15 women treated surgically for invasive breast cancer 12–18 months prior to recruitment (2020). 24 women with breast cancer and 24 members of staff (2023).	• Exploring patients’ experiences of completing the Macmillan eHNA (2020). • Exploring perceptions of the Macmillan eHNA, including how the eHNA was completed from the perspective of staff and patients (2023).
	** Snowden et al. 2023** Scotland Randomised controlled trial across four outpatient oncology clinics	To establish whether incorporating HNA into consultations would increase patient participation, shared decision making and post consultation self-efficacy.	Head and Neck, skin or colorectal cancer 147 adults with confirmed diagnoses attending follow-up consultations 3 months post-treatment between 2015 and 2020.	• Self-reported HNA Concerns Checklist completed on paper. • Assessing emotional, practical, financial and/or clinical concerns.
	** Snowden et al. 2024** Scotland Controlled observational retrospective cohort study collecting data held with hospitalisation, cancer registration prescription and unscheduled care service data held by Public Health	To establish whether the Improving Cancer Journeys (ICJ) service users used less unscheduled care in comparison to a matched sample of controls.	Bowel, breast, lung, prostate and ‘other’ 1214 adult users of the Improved Cancer Journey (ICJ) in Scotland.	• ICJ service including HNA and Distress Thermometer and Care Planning tools as part of the Improving Cancer Journeys services • Cancer registrations linked to: Ø NHS24 calls Ø A&E admissions Ø Inpatient hospital admissions Ø Unscheduled care Ø Number & cost of psychotropic prescriptions
	**Le Boutillier et al. 2023** London, England Prospective cohort study	To use the Adversity, Restoration and Compatibility (ARC) framework to underpin the HNA to improve the experience of personalised care and support planning for people living with treated but not cured (TbnC) cancer.	Treatable, but not curable (TbnC), cancer (metastatic breast, lung, colorectal myeloma). 51 patients receiving care from adult ICHT services on a TbnC cancer pathway, 6–24 months postdiagnosis.	• Macmillan HNA used to underpin the implementation of the Adversity-Restoration-Compatibility (ARC) clinic, as part of a quality improvement project • HNA delivered over three Allied Health Professional led interactions comprising: Ø initial telephone contact with each patient to offer the ARC clinic invitation; Ø face-to-face outpatient appointment; Ø follow-up telephone consultation offered after four weeks to review patients’ goals
Palliative care needs assessment tool (PC-NAT)	** Lambert et al. 2018** New South Wales, Australia Qualitative study with an interrupted time series	To examine (1) approaches used by oncologists to administer the palliative care needs assessment tool (PC-NAT) in consultations with patients with advanced cancer and their caregivers, (2) potential of this tool to facilitate discussion of psychosocial issues, and (3) whether use of the tool alters the length of consultations.	Advanced cancers (breast, bowel, myeloma, lymphoma, melanoma, leukaemia, gynaecological, prostate, stomach and bladder). Main study—219 adult outpatients diagnosed with advanced cancer. Sub-study—45 potentially eligible patients of which 20 consented for their consultations.	• One page PC-NAT comprising five sections to assess patients’ needs: Ø Three items to determine need for further assessment; Ø Seven items to assess level of concern with the patients; Ø Five items to assess the healthcare professionals’ level of concerns with the caregiver’s ability to care for the patient; Ø Two items to assess the caregiver’s well-being; Ø One item to assess whether the HCP thought the patient needed referral to another service
Sheffield Profile for Assessment and Referral to Care (SPARC) questionnaire	** Wilcock et al. 2019** Nottingham, England Cross sectional survey in Rehabilitation service in Nottingham University NHS Trust	To identify the most common and/or distressing supportive and palliative needs present soon after diagnosis using a specifically developed questionnaire.	Thoracic 738 adult patients three to six weeks after cancer diagnosis.	• SPARC questionnaire completed to identify multiple physical and psychological symptoms over a 26-month period.

### Quality of studies

Quality assessment was conducted on all studies using the Specialist Unit for Review Evidence (SURE) [34] critical appraisal checklist (see Table 3) with varying quality. One study reported on a randomised controlled trial focusing on specific cancers across two clinics within a specialist cancer centre and two clinics located in general hospitals [23]. The remaining studies comprised a range of study designs. Three studies were cross-sectional in design [38, 40, 42], all of which did not meet all quality requirements. Three studies adopted a qualitative approach through semi-structured interviews of which two met the requirements to be classified as high quality [36, 37] and the third which was a sub-study from an interrupted time series study, comprising audio-taped consultations, did not meet the requirements of being of high quality [43]. The other studies were based on a controlled observational retrospective cohort study [41], and a quality improvement project focused on the implementation of the HNA using the Adversity, Restoration, Compatibility (ARC) framework rather than the HNA tool itself [39]; neither study met full requirements to being of high quality.

**Table 3:**
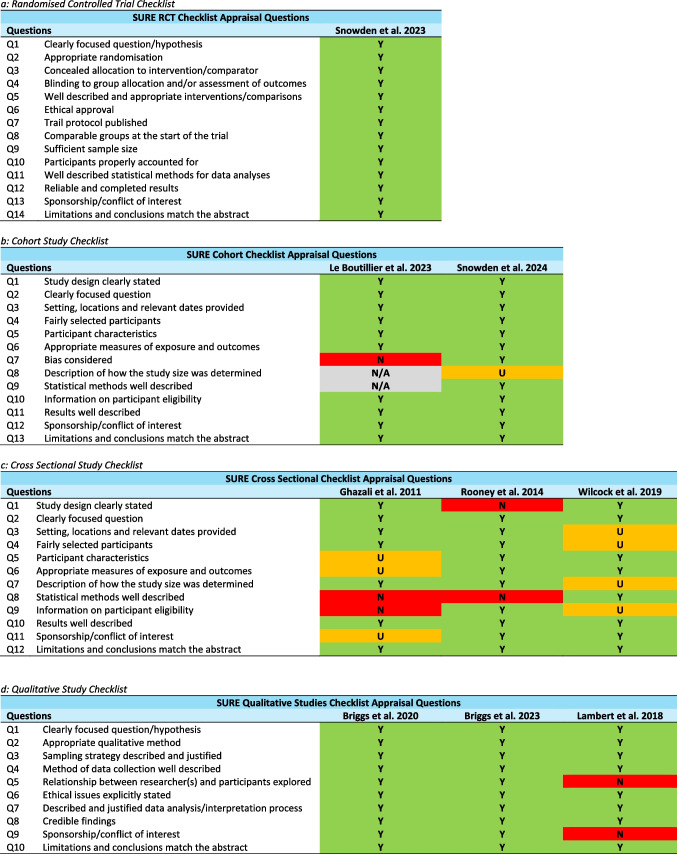
Quality assessment of the included studies

### Tools to identify HNAs in cancer

Table 4 outlines the results of the study outcomes relating to the implementation of the HNA tools.

**Table 4:** Study outcomes relating to implementation

HNA	Study	Implementation	Identified needs/concerns	Patient participation and shared decision-making
The Liverpool Patients Concerns Inventory (PCI)	** Rooney et al. 2014**	**Acceptability (patient):** 80% (*n* = 36/45) reported completing the PCI by themselves, with 60% completing the PCI in under 15 min. The remaining PCIs were completed by the patients’ main caregivers. After the clinic, patients were invited to fill out a ‘final feedback’ form. The final feedback form was completed by 32/53 (60%) patients. Ninety-four per cent of patients reported the PCI to be either ‘useful’ or ‘very useful’ (*n* = 30/32) 43% (*n* = 23/53) completed the PCI in advance of the clinic and were in a range of between 1–12 months from the end of their primary therapy. 85% (*n* = 45/53) completed the initial feedback form. **Acceptability (staff):** (*n* = 19/21, 91%) of staff viewed the PCI as useful and almost all (*n* = 20/21, 94%) reported that the PCI aided communication with patients. However, PCI consultations were perceived to last longer (*n* = 14/21, 67%).	94% (*n* = 50/53) had at least one concern; the median number of concerns identified was eight (range 0–36). Over a quarter of patients reported more than 17 concerns; of these high-frequency concerns, nine are either not listed or only partially addressed on the NCCN DT (concerns regarding memory, fear of tumour coming back, concentration, headache, weakness in arms or legs, vision, personality changes, coordination, and speech).	25% (*n* = 13/53) of patients expressed a wish to be referred to another specialty, most frequently physiotherapy (*n* = 5/53, 9%).
	** Ghazali et al. 2011**	**Acceptability (patient):** The authors declare that despite patients being older (36% of men and 56% of women were above 65 years of age), this did ‘not seem to hamper’ the use of the touch screen computer, however there is no data included in the results to support quantify this. **Feasibility:** Introduction of the PCI did not change prevalence of new onward referrals.	No change in the overall prevalence of onward referral following PCI. Referrals to oral rehabilitation and psychological support increased following introduction of the PCI Consistent (high) request for referral to speech and language, and dentistry services.	Not reported.
Macmillan’s Holistic Needs Assessment (HNA)	** Briggs et al. 2020**	**Acceptability:** *n* = 3/15 reported the eHNA to be helpful.	Each participant discussed the physical symptoms they had experienced and appeared to be more forthcoming in divulging this information than when discussing the emotional aspects of cancer diagnosis and care.	Participants were reluctant to raise psychological issues compared with physical issues. Several participants described concerns around feeling able to raise psychological issues and a fear of facing hospital readmissions if demonstrating an inability to cope.
	** Briggs et al. 2023**	**Acceptability (patients):** *n* = 13/15 women reported that the eHNA either was not useful or had minimal benefit to them. **Acceptability (staff):** For participants with a nursing background, many felt that the eHNA presented a threat to their role, as use of the tool suggested they were not undertaking their jobs effectively. **Fidelity:** Many staff indicated uncertainty around the ‘correct’ way to conduct an eHNA. **Adoption:** Organisational hierarchy or culture within staff structures appeared to influence how successfully the eHNA was implemented, with some staff being resistant to change.	Participants reported not being confident to identify which needs should be reported to clinicals staff.	Several participants reported a lack of confidence in completing an eHNA and knowing which concerns were appropriate to raise with staff.
	** Snowden et al. 2023**	**Feasibility:** Consultation times were on average nearly 2 min longer for the intervention group (17 min 25 s) than control group (15 min 39 s).	Not reported.	Patient Initiative scores were lower in the intervention group (0.25 ± 0.10) than control group (0.26 ± 0.13), a non-significant difference of − 0.012 (95% CI − 0.06 to 0.04), t(145) = − 0.439, p = 0.661). Dialogue Ratio scores were lower for the intervention group (0.38 ± 0.15) than control group (0.40 ± 0.19), a nonsignificant difference of − 0.013 (95% CI − 0.05 to 0.02), t(145) = − 0.691, p = 0.49. CollaboRATE scores (Shared decision making) were higher for the intervention group (25.43 ± 4.1) than control group (25.03 ± 4.4), a non-significant difference of 0.4 (95% CI − 1.77 to 0.97), t(149) = − 0.58, p = 0.562.
	** Snowden et al. 2024**	**Feasibility:** A&E (attendance one or more times) Baseline • 27% of ICJ users • 27% in Glasgow • 26% in rest of Scotland Study Period • 41% of ICJ users • 25% in Glasgow • 24% in rest of Scotland Hospital attendance (one or more admissions) Baseline • 43% of ICJ users • 427% in Glasgow • 33% in rest of Scotland Study Period • 77% of ICJ users • 68% in Glasgow • 67% in rest of Scotland Unscheduled care pathways (one or more recorded) Baseline • 46% of ICJ users • 42% in Glasgow • 42% in rest of Scotland Study Period • 61% of ICJ users • 51% in Glasgow • 53% in rest of Scotland Prescriptions for psychotropic drugs (one or more prescriptions) Baseline • 95% of ICJ users • 94% in Glasgow • 99% in rest of Scotland Study Period • 99% of ICJ users • 98% in Glasgow 98% in rest of Scotland	Not reported.	Not reported.
	**Le Boutillier et al. 2023**	**Acceptability (patients):** *n* = 62/113 eligible patients declined to take part. **Feasibility:** Compared with routine initial HNA there was an increase in completion of the care plans from 13 to 90% after use of the ARC HNA. HNA assessment was an additional series of appointments on top of their usual care conducted by AHPs. 54.9% of eligible patients in the implementation study declined to take part in the study (*n* = 62/113) due to a range of reasons; break down per cancer:] • 71% breast • 69% prostate • 60% myeloma groups and tended to report they were ‘doing well’, and therefore, did not need this input (44.8%) There was a higher level of uptake was noted in the lung group (61.1%).	An average of 12 new concerns were identified per patient compared with their initial HNA, and 96% of patients achieved at least one of their goals. 37% of patients were referred to start new rehabilitation interventions and 53% were referred to supportive care services	63 new goals set by patients in the initial ARC clinic visit including walking (28%), sedentary leisure (24%), exercising (16%) and instrumental activities of daily living (14%). All care plans shared with general practitioners. Patients reported that they valued the space for reflection and follow-up, and clinicians valued the collaborative approach to meeting patients’ supportive care needs.
Palliative care needs assessment tool (PC-NAT)	** Lambert et al. 2018**	**Feasibility:** There was no significant difference in the mean length of the PC-NAT consultations (18.1 min,) and the baseline consultations (19.5 min). **Fidelity:** Mode of administration of the PC-NAT was not optimal to identify and discuss the psychosocial concerns of patients and their caregivers. Oncologists most often administered the PC-NAT in the final third of the consultation and integrated into the consultation. Majority of interactions between the clinician and patient related to medical and treatment issues, regardless of whether the PC-NAT was used or not and with little to no discussion of broader psychosocial concerns. No significant difference in the mean length of the PC-NAT consultations (18.1 min, range = 3.5–53.51) and the baseline consultations (19.5 min, range = 9.5–24.1).	Interactions between the oncologist and patients related to medical and treatment issues, and with little to no discussion of broader psychosocial concerns.	Oncologists tended to respond to patients’ and caregivers’ psychosocial concerns with a lower level of empathy than medical concerns.
Sheffield Profile for Assessment and Referral to Care (SPARC) questionnaire	** Wilcock et al. 2019**	**Acceptability (patient):** Completion rates were generally high. The commonest known reasons for questionnaire noncompletion were being too unwell/death (*n* = 125, 17%) and residing outside of the rehabilitation service area (*n* = 98, 9%). Respondents were able to leave the question blank if they did not know how to answer the question; the highest proportion of blank responses occurred in the treatment Sect. (30%), mostly as patients had yet to commence treatment.	The proportion of patients reporting at least one symptom or issue causing ‘‘very much,’’ ‘‘quite a bit,’’ or ‘‘a little’’ distress or both were 67%, 88%, and 99%, respectively. The median (IQR) number of symptoms or issues was 15 (11–21), ranging from 0 to 41. Two psychological symptoms were reported by 67% of patients: worrying about effects of the illness on others and feeling anxious.	Many patients wanted further information: • 21% wanted more information about their condition; • 30% wanted more information about their care, or treatment; • 24% wanted more information on financial issues.

Two studies used the Liverpool PCI, which provides patients the opportunity to complete a tick list of potential needs and areas that patients would like to discuss and/or be referred to including physical and functional well-being, treatment, social care and social well-being, psychological, emotional, and spiritual well-being [38, 40]. One of these studies included patients with head and neck cancer [38]. The other study included patients with brain cancer [40].

Five studies [23, 36, 37, 39, 41] used Macmillan’s HNA which provides a checklist that patients can identify any physical, practical, emotional, family or relationship, spiritual, and information or support needs along with any questions they may want to be addressed by their relevant clinician. Two studies [38, 39] used the eHNA in women with breast cancer. Snowden et al. [41] used the paper version of the HNA Concerns Checklist in patients with head and neck, skin, or colorectal cancer. Another study [41] also reported on the use of the HNA and the NCCN DT and Care Planning tools as part of the Improving Cancer Journeys (ICJ) services in patients with bowel, breast, lung, prostate, and ‘other’ cancer. In addition to this, the Macmillan HNA was used to underpin the implemented ARC clinic, as part of a quality improvement project for patients with treatable but not curable cancer (metastatic breast, lung, colorectal myeloma); patients 6–24 months postdiagnosis were eligible [39].

One study used a one-page Palliative Care Needs Assessment Tool (PC-NAT) in patients with advanced cancer (including breast, bowel, myeloma, lymphoma, melanoma, leukaemia, gynaecological, prostate, stomach and bladder cancers) [45]. One study used the SPARC questionnaire in patients with thoracic cancer [42].

Table 4 provides details on the outcomes focusing on implementation of the HNAs, patients’ needs and concerns, and patient participation and shared decision-making.

### Implementation and impact of HNAs in cancer

#### Acceptability

Acceptability was the most common implementation outcome category used in the included studies [36–40, 42]. The range of outcomes relating to the acceptability of the HNA tools varied, including patient and staff satisfaction [36, 39, 40], participation and completion of the tool [39, 41, 42] as well as the acceptability of the format [38].

#### Feasibility

Five studies reported on assessing the feasibility of the HNAs [23, 38, 39, 41, 43]. One study looked at the number of new onward referrals from using the PCI and reported there to be no change [38]. Another study reported an increase in completion of the care plans from 13 to 90% after use of the ARC HNA [39]. Two studies [41, 43] reported on the length of the consultations; one study reported no significant difference in the mean length of the PC-NAT consultations (18.1 min) and the baseline consultations (19.5 min) [41], whereas another study reported an increase in the duration of the consultations using the HNA; consultation times were on average nearly 2 min longer for the intervention group (17 min 25 s) than control group (15 min 39 s) [23].

One study reporting on the use of healthcare services including calls to NHS24 (Scotland’s national telehealth and telecare service, providing a telephone advice and triage service), access to the Accident and Emergency (A&E) department, attendance to hospital, unscheduled care pathways and prescriptions for psychotropic drugs for both the Improving Cancer Journeys (ICJ) group [41]. Overall, it reported an increase from baseline through the study period for all groups; however, there was a greater use of the healthcare services by participants in the ICJ group [41]. There was a statistically significant increase in the number of NHS24 calls when comparing it with the Glasgow group *d* = 0.39, *p* = 0.0016) and a non-statistically significant increase when comparing with the Rest of Scotland (*d* = 0.01, *p* = 0.84) [41]. There was a statistically significant increase in attendance to A&E when comparing to the Glasgow (*d* = 0.19, *p* < 0.0001) and Rest of Scotland groups (*d* = 0.11, *p* = 0.0013) [41]. There was also a statistically significant increase in the number of hours spent in A&E when comparing to the Glasgow (*d* = 1.38, *p* < 0.0001) and Rest of Scotland groups (*d* = 1.28, *p* =  < 0.0001) [41]. There was a statistically significant increase in the number of admissions to, and days spent in the hospital when comparing with the Glasgow (admissions: *d* = 1.66, *p* < 0.0001; days in hospital: *d* = 3.68, *p* < 0.0001) and the Rest of Scotland groups (admissions: *d* = 3.68, *p* < 0.0001; days in hospital: *d* = 3.41, *p* < 0.0001) [41]. There was a statistically significant increase in the number and complexity of care pathways (number of steps) when comparing with the Glasgow (number of pathways: *d* = 0.38, *p* < 0.0001; complexity of care pathway: *d* = 0.9, *p* < 0.0001) and the Rest of Scotland groups (number of pathways: *d* = 0.19, *p* = 0.041; complexity of care pathways: *d* = 0.65, *p* < 0.0001) [43]. There was a non-statistically significant increase in the number of psychotropic drug prescriptions and cost of prescriptions when comparing with the Glasgow group (number of prescriptions: *d* = 0.32, *p* = 0.21; cost of prescriptions: *d* = −0.061, *p* = 0.99) [43]. There was a statistically significant increase when comparing this with the Rest of Scotland group (*d* = 0.67, *p* = 0.0075) and a non-statistically significant increase in cost of prescriptions (*d* = −0.061, *p* = 0.99) [43].

#### Fidelity and adoption

Other outcomes reported on included fidelity [37, 43] and adoption [37]. Fidelity can be defined as ‘the degree to which an intervention was implemented as it was prescribed in the original protocol or as was intended by programme developers’ [13]. One study reported on the mode of the PC-NAT administration; oncologists most often administered the PC-NAT in the final third of the consultation, integrating it into the discussion with little preamble regarding its purpose or significance [43]. Analysis of the consultations revealed that most interactions between the clinician and patient related to medical and treatment issues, regardless of whether the PC-NAT was used or not; there was little to no discussion of broader psychosocial concerns [43]. There was no significant difference in the mean length of the PC-NAT consultations (18.1 min, range = 3.5–53.51) and the baseline consultations (19.5 min, range = 9.5–24.1) [43].

One study found that many staff indicated uncertainty around the ‘correct’ way to conduct an eHNA or how to address concerns when no clear solution was available [37]. Views varied toward the benefits and burdens of the assessment and its implementation, but the existence of target eHNA completion metrics presented pressure and ‘feelings of obligation’, which affected quality of assessments for some staff [37]. Lastly, organisational hierarchy or culture within staff structures appeared to influence how successfully the eHNA was implemented, with some staff being resistant to change [37]. Participants’ perceptions of the eHNA appeared contingent on the organisational structure and processes within which it was situated [37]. How the organisation approached eHNA implementation seemed connected to staff views toward it. This included whether an emphasis was placed on achieving targets around eHNA completion and the engagement and support from senior leadership [37].

### Identified needs/concerns

Seven studies reported needs and concerns identified by patients and/or carers [38–45]. The aims of the HNA interventions in the included studies varied. Some allowed for patients to indicate their needs or concerns that they wanted to discuss in a clinical consultation [38, 40]; to document the referral process of patients following their consultation in an outpatient clinic and compared the referral trends before and after introduction of the intervention [38]; and to identify new concerns in an initial visit to the interventional clinic, the new goals and maintained goals made over time and identify onward referrals [39]. One study found that participants were more likely to raise psychological issues rather than physical concerns [36]. Participants also reported not being confident to identify which concerns need to be reported with staff [37]. Another study reported little interactions between the oncologist and patients when referring to areas of medical and treatment concerns; there was little to no discussion of any broader psychosocial concerns [43].

### Patient experience of HNA and shared decision-making outcomes

Different approaches to assessing patient participation in the implementation of the HNA interventions were reported. One study found some participants described concerns around feeling able to raise psychological issues and several participants reported a fear of facing hospital readmission if they demonstrated an inability to cope [36]. One study reported the perspective from health care professionals, reporting oncologists’ response to patients’ and caregivers’ psychosocial concerns with a lower level of empathy than medical concerns [43]. Another study reported an increase in completion of the care plans when compared with the initial ARC HNA (from 13% of completion to 90% of completion) [39] and reported that they valued the space for reflection and follow-up, and clinicians valued the collaborative approach to meeting patients’ supportive care needs [39]. One study recorded patients’ request for referrals to other specialities; 25% (13/53) of patients expressed a wish to be referred to another specialty, this was most frequently physiotherapy (*n* = 5/53, 9%) [40]. One study looked at requests for information via their HNA [42]: 21% wanted more information about their condition; 30% wanted more information about their treatment; and 24% wanted more information on financial issues [42]. One study assessed patient participation within the consultation [23], reporting that the HNA did not change the amount of conversation initiated by the patient or the level of dialogue within the consultation [23]. Furthermore, the HNA did not change patients’ sense of collaboration or feelings of self-efficacy afterwards [23].

## Discussion

There is a paucity of evidence of HNAs being successfully implemented in brain tumour and the wider cancer clinical settings. The studies included in this review demonstrated a range of different approaches to evaluate the acceptability, feasibility, fidelity and adoption of the HNA tools. However, there was a lack of consensus on the most appropriate timing, location and method of HNA delivery. There was also variation in patients’ needs and concerns being raised via HNA and how patients experienced using the HNA in the reported studies.

Despite there being some evidence for the implementation of HNAs in the wider cancer clinical settings, the evidence is both limited and lacked consistency in the approaches to implementation in practice. Furthermore, Snowden et al. found that the use of the HNA, the NCCN DT and Care Planning tools as part of the ICJ services does not lead to a decrease in unscheduled care, hospitalisation nor in the cost of medications [41]. The wider structuring of services incorporating HNA such as in the above study is linked to a wider report evaluating ICJ in Scotland, funded by Macmillan [46]. One study [43] reported results which were contradictory to their hypotheses that use of the ICJ would result in a reduction of use and access to health care services. Taking a holistic approach to assessing patients’ needs, these factors are likely to be complex and may, in part, be related to wider factors such as improvement in access to care and raised awareness of availability of relevant services [41]. In the wider evidence, other systematic reviews report similar limitations. In 2019, a systematic review consisting of a thematic synthesis of the implementation and impact of HNAs for people with cancer identified 21 studies (including randomised controlled trials, service evaluations and feasibility studies) with limited evidence of the impact of use of HNAs on patient outcomes [45]. These studies often reported on the percentage-change of symptoms, with inconsistency in the purpose of the interventions and a lack of evidence of ‘post-assessment downstream care’ [45]. This inconsistency in implementing instruments to assess needs can also be found when supporting the needs of caregivers of cancer patients. A systematic review of caregivers for cancer patients receiving palliative care also reported limited and inconsistent content, constructs and how the tools were assessed and applied in practice [46].

We identified one brain tumour specific HNA tool which was designed to be more sensitive to the needs and concerns of brain tumour patients than general cancer checklists [40]. A secondary analysis of a prospective cohort study in high grade glioma reported a stepped allocation of four levels of needs for triaging patients to the relevant support [47, 48]. Screening tools were reported to be most effective if used within clinical pathways to identify unmet needs; this approach can inform and direct the provision of support and improve quality of life [48]. While several generic HNAs in cancer settings exist and address a broad range of concerns, their value for brain tumour patients is uncertain, particularly considering the neurocognitive impacts, specific physical symptoms such as headache and epilepsy, burdens of treatment and impacts on social functioning which affect patients from the early stages of the illness [4]. The limited and lack of consistency of HNAs implemented in wider clinical settings also restrict the ability to provide clarity and guidance on the use of HNAs in routine clinical practice. The wider evidence describes the challenges in implementing HNAs in the brain tumour population. In 2019, one study evaluated health-related quality of life patient-reported outcome measures used in supportive care interventions for people with brain tumour, their psychometric properties and clinical utility [28]. This study identified four tools which had limited psychometric validation, and a lack of brain tumour-specific HNAs [28]. In 2019, Roselund et al. reviewed the use of patient-reported outcomes for high-grade glioma, in both trials and clinical practice [49]. Two tools were identified for possible use in the clinical settings and discussed potential difficulties of implementing these tools in clinical practice: an HNA tool using the NCCN DT and a supportive care needs tool [49]. While the HNA may address the complexities of glioma, patient-reported outcome measures to improve long-term care in routine clinical practice was reported to be lacking [49].

### Study strengths and limitations

The main strength of this review was the comprehensive inclusion of any study design, which explicitly included implementation and/or reported outcomes. Furthermore, the decision to undertake a systematic review highlighted not only the lack of evidence in brain tumour but also in the wider cancer setting. The evidence revealed small sample sizes and heterogeneous study designs including intervention type, timing, and outcome measurements, precluding any definitive recommendations. This also highlighted the limited specific interventions to support holistic assessments which could be tailored to be used for people with brain tumour in clinical care settings, although the one study we identified did indicate potential for use more widely [42]. This review does not highlight a lack of HNAs being used in clinical practice per se, rather indicates a limitation in the evidence-base being implemented and published in the academic literature. Furthermore, this indicates the potential for inconsistencies and limited clarity on which HNAs can be used for the brain tumour population.

## Conclusion and future directions

There is limited evidence of HNAs being successfully implemented in brain tumour and the wider cancer healthcare settings. Tailored interventions for the needs of people with brain tumour, implemented at key time points, has the potential to address the complexities they face. However, clear evidence-based guidance to develop relevant HNAs for successful implementation in clinical settings is needed. Insight into what outcomes, how they are used, when they need to be used and by whom to assess the needs of people with brain tumour in routine clinical practice is needed to develop and implement consistent, appropriate tools to improve patient care.

## Data Availability

Additional search strategies, critical appraisal checklists, data extraction forms, and list of excluded studies are available on request.
